# Reaching the limit

**DOI:** 10.7554/eLife.39804

**Published:** 2018-08-10

**Authors:** Benedetta Bolognesi, Ben Lehner

**Affiliations:** 1 Systems Biology ProgramCentre for Genomic Regulation, Barcelona Institute of Science and TechnologyBarcelonaSpain; 2 Institute of Bioengineering of CataloniaBarcelona Institute of Science and TechnologyBarcelonaSpain; 3 Universitat Pompeu FabraBarcelonaSpain; 4 Institució Catalana de Recerca i Estudis AvançatsBarcelonaSpain

**Keywords:** protein burden, overexpression, glycolysis, *S. cerevisiae*

## Abstract

How many copies of a protein can be made before it becomes toxic to the cell?

**Related research article** Eguchi Y, Makanae K, Hasunuma T, Ishibashi Y, Kito K, Moriya H. 2018. Estimating the protein burden limit of yeast cells by measuring expression limits of glycolytic proteins. *eLife* **7**:e34595. doi: 10.7554/eLife.34595

Cells can be pictured as factories that build proteins, the molecules essential for nearly all of life’s processes. The body tightly controls production levels, because creating too many proteins – also known as protein overexpression – can be harmful to the cell. Yet, it is difficult to know how much of any given protein will be harmful, or why.

Indeed, high concentrations of enzymes and other proteins can harm cells in several ways, for example by activating or overloading specific biological pathways, disrupting regulation, or by aggregating together (Vavouri et al., 2009; Tang and Amon, 2013; Makanae et al., 2013). They can also upset the balance in protein complexes or make the different liquid phases separate in the cell (Birchler and Veitia, 2012; Bolognesi et al., 2016). Ultimately, overexpressing any protein will be destructive because it exhausts the resources of the cell to make and transport proteins (Stoebel et al., 2008). However, we did not know how much of a specific protein must be produced to cause this ‘protein burden’ and hinder cell growth.

Now, in eLife, Hisao Moriya and colleagues at the universities of Okayama, Kobe and Meiji – including Yuichi Eguchi as first author – report that many members of a group of enzymes can be overexpressed until they form 15% of the total proteins in a yeast cell (Eguchi et al., 2018). Only then do they start to cause damage because of protein burden. This matches the results of previous experiments from the same laboratory, which only focused on a single fluorescent protein that did not interfere with any components of the cell (Kintaka et al., 2016).

To discover this limit, Eguchi et al. relied on a method the lab developed in 2006. The technique involves inserting a small portion of DNA, called a plasmid, into the yeast cells. The plasmid carries two genes: the first is essential for growth, and the other codes for one of the enzymes studied. The cell increasingly needs to make new plasmids in order to grow, but this also creates more enzymes. In this ‘tug-of-war’ system, the yeast generates more and more plasmids until the expression of the enzyme of interest becomes harmful; at this point, plasmid production decreases. The number of plasmids in the cell thus reflects the quantity of protein that can be made before it turns toxic.

The experiments focused on a set of 29 glycolytic enzymes, which break down sugar in yeast. These enzymes are normally highly expressed in a cell, and their roles are well understood.

Out of the 29 proteins, three were not harmful in the experiment and could not be produced in high enough amounts to reach the burden limit. This was because the genes that encoded these enzymes contained sequences that were not optimal for protein production.

Another 19 enzymes could be expressed until they formed close to 15% of the total protein content of the cell, which suggests that protein burden is the cause of their toxicity. The fact that even large essential yeast enzymes could be produced up to this limit is unexpected, and it suggests that in many cases the toxicity created by protein overexpression does not depend on the specific characteristics of the proteins.

The cost of overexpression may come from the burden it puts on the machinery that assembles proteins in the cell, which requires particularly high levels of energy (Shah et al., 2013). Putting this apparatus under pressure could impair or slow it down; in turn, this may hinder the creation of other proteins and decrease the fitness of the cell. The other steps of protein production, such as ‘reading’ the genes, helping the protein to mature, bringing it to its right location in the cell, and degrading it, also use significant amounts of energy (Rice and McLysaght, 2017).

Seven proteins caused harm at concentrations far below the 15% limit, which means that they must damage the cell in other ways than by causing a protein burden. Eguchi et al. identified three mechanisms for this toxicity: the proteins aggregated together, they overloaded a transport system that takes them to a specific cell compartment, or the overexpressed enzymes produced too much catalytic activity (Figure 1). One might have expected this last process to drive the toxic effects of this group of proteins. Yet, killing catalytic activity in the enzymes (by introducing specific mutations) only relieved the toxicity caused by overexpression for two of the 18 proteins that were tested.

**Figure 1.**
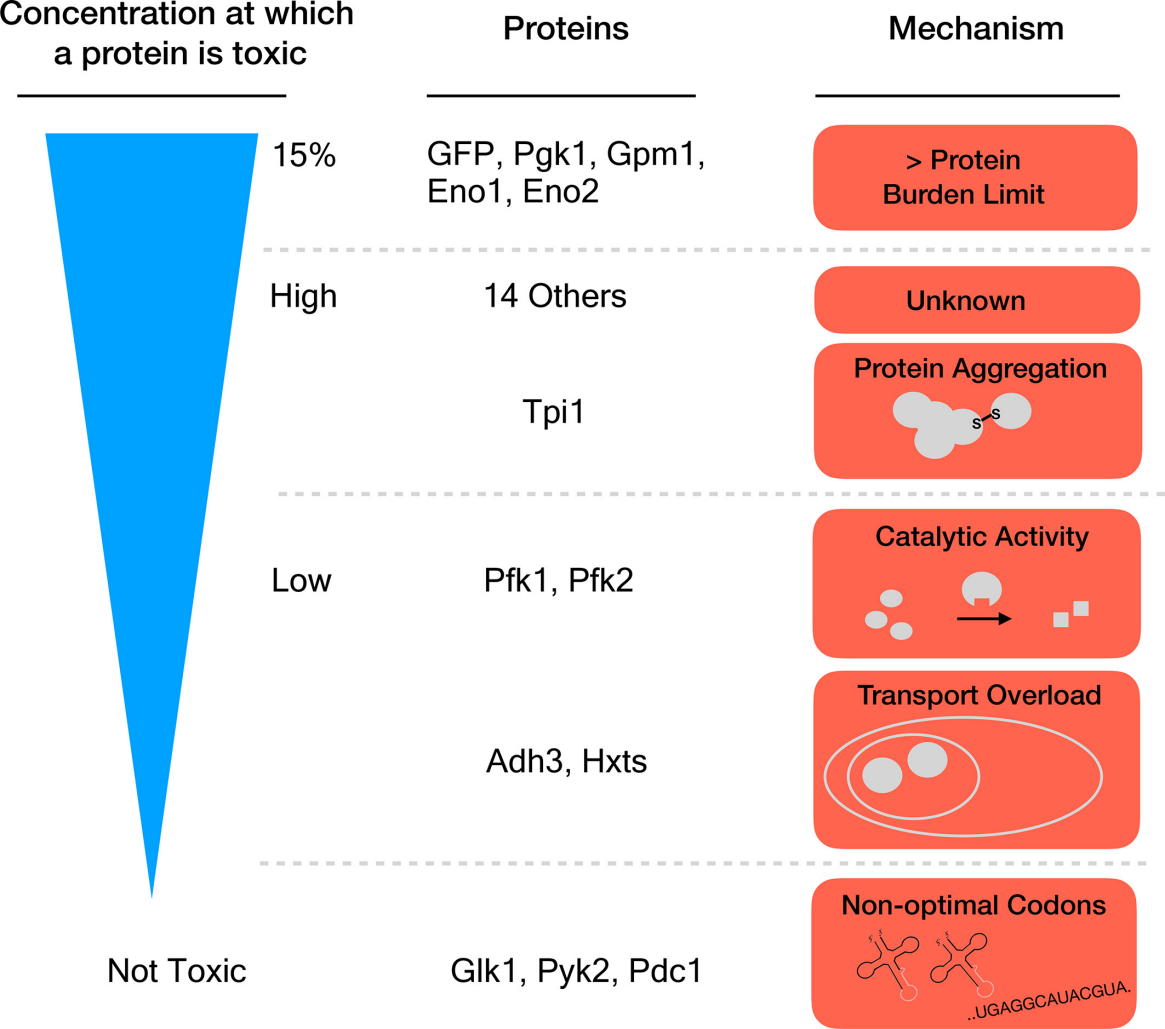
Different mechanisms of toxicity induced by protein overexpression. Many enzymes involved in glycolysis such as GFP or Pgk1 do not cause any harm until they are overexpressed up to or close to the protein burden limit, which corresponds to 15% of the total proteins in the cell. Proteins that are toxic before reaching this limit cause harm via mechanisms other than the exhaustion of cellular resources. For example, while Tpi1 can still be expressed at relatively high levels (close to 15%), it causes protein aggregation. Enzymes such as Pfk1 or Adh3 can only be expressed at lower levels before they are toxic: Pfk1 causes too much catalytic activity while Adh3 overloads transport systems. Some proteins, for example Glk1, Pyk2 and Pdc1, are not harmful when overexpressed because they simply cannot reach the protein burden limit. Expression of these genes is lower because they use rare codons (sequences that are less optimal for protein production).

In many cases, removing one mechanism of toxicity increased the level to which an enzyme could be overexpressed, but it still did not allow expression up to the 15% limit. Proteins could therefore be damaging through a range of mechanisms, each of which gets triggered when the concentration in the cell reaches a particular level.

While the glycolytic enzymes belong to the same pathway and share extremely similar roles, their overexpression affects cell growth via diverse mechanisms. In other words, the biological role of a protein cannot be used to predict how it will harm the cell. Altogether, these results stimulate important lines of enquiry, such as looking into which of the above mechanisms damage cells when gene expression changes during disease. They also encourage further research so that we could predict at which concentration the expression of every human gene will be harmful in any tissue. And finally, they raise the question: is protein burden what has stopped increased gene expression during evolution?
